# Mitigating High-risk EBV and CMV Through Kidney Paired Donation: A Survey of Potential Donor and Recipient Candidates

**DOI:** 10.1097/TXD.0000000000001737

**Published:** 2024-11-15

**Authors:** Arjun Kalaria, Rajil B. Mehta, Puneet Sood, Xingyu Zhang, Harry J. Morford, Vishnu Potluri, John F. P. Bridges, Chethan M. Puttarajappa

**Affiliations:** 1 Renal-Electrolyte Division, Department of Medicine, University of Pittsburgh Medical Center, Pittsburgh, PA.; 2 Department of Surgery, Thomas E. Starzl Transplantation Institute, University of Pittsburgh Medical Center, Pittsburgh, PA.; 3 Division of Nephrology, Department of Medicine, University of California, San Francisco, CA.; 4 School of Health and Rehabilitation Sciences, University of Pittsburgh, Pittsburgh, PA.; 5 Renal-Electrolyte and Hypertension Division, Hospital of the University of Pennsylvania, Philadelphia, PA.; 6 Department of Biomedical Informatics, The Ohio State University College of Medicine, Columbus, OH.

## Abstract

**Background.:**

High-risk cytomegalovirus (CMV) and Epstein-Barr virus (EBV) mismatches (ie, seropositive donors to seronegative recipients) among kidney transplant recipients lead to increased healthcare utilization, inferior allograft outcomes, and high mortality. We assessed the interest among prospective kidney donor and recipient candidates to participate in kidney paired donation (KPD) for averting CMV/EBV high-risk mismatches.

**Methods.:**

We surveyed 51 potential living donors and 102 kidney recipient candidates presenting for their evaluation visit at the University of Pittsburgh Medical Center between October 2022 and May 2023. We evaluated their general inclination toward KPD and their interest in KPD under various risk-benefit scenarios, particularly emphasizing the mitigation of high-risk mismatches associated with EBV and CMV. This was done using a 5-point Likert scale (1-low interest; 5-high interest) customized survey.

**Results.:**

There was high interest in KPD among both donor and recipient candidates (median score 4 versus 4; *P* = 0.09). However, donor candidates had a lower interest in KPD if they were compatible with their intended recipient (median score 2 versus 4; *P *< 0.001). Most donor (80.4%; N = 41) and recipient candidates (89.2%; N = 91) expressed a strong willingness to participate in KPD to prevent high-risk CMV and EBV mismatches, but this interest declined with longer transplant delays. Interest also varied on the basis of participants’ income and employment status.

**Conclusions.:**

Interest in KPD for avoiding CMV and EBV was high among both donor and recipient candidates. Additional research is required to assess the capacity and desirability for KPD expansion, particularly among ABO and HLA-compatible pairs.

## Graphical abstract

**Figure:**
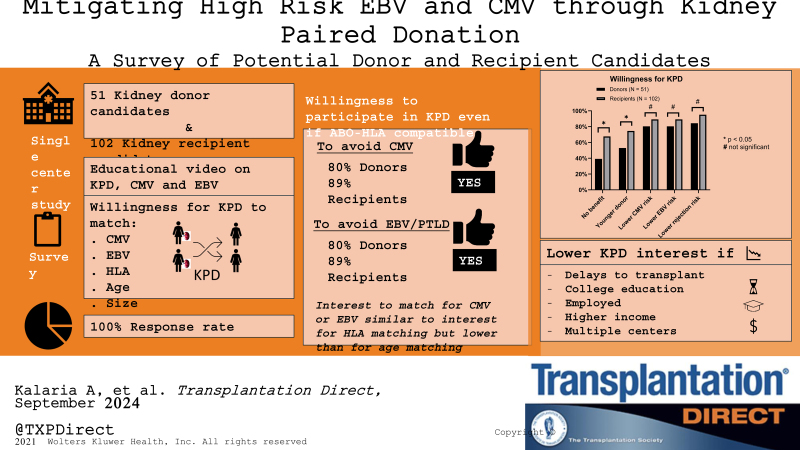

Epstein-Barr virus (EBV) and cytomegalovirus (CMV) infections among kidney transplant recipients (KTRs) increase morbidity and mortality.^1-10^ This risk is particularly high when recipients without prior exposure to these viruses receive a kidney from a donor with prior CMV or EBV infection (ie, donor IgG positive, D^+^, to recipient IgG negative, R^–^). Despite antiviral prophylaxis, 20%–30% of high-risk CMV-mismatched KTRs develop CMV infections after stopping prophylaxis.^1,2^ CMV infections increase hospitalizations and healthcare costs and negatively impact outcomes after KT. Population studies have shown a higher risk of graft loss and mortality among KTRs with high-risk CMV mismatches.^4,5^ Similarly, high-risk EBV status is a major risk factor for the development of posttransplant lymphoproliferative disorder (PTLD), which often necessitates therapy with chemotherapeutic agents.^8-10^ Despite improvement in PTLD treatment over time, patients have 4–10 times higher mortality risk, particularly in the first few years after PTLD diagnosis.^8-10^ While therapies to manage CMV and EBV infection-related complications are expanding, including newer antivirals and adoptive T-cell therapies, they come with additional costs and side effects. Therefore, there is interest in alternative management strategies, such as vaccinations and the use of donor-recipient matching to avoid high-risk mismatches. Given the current lack of approved CMV or EBV vaccines, pretransplant CMV and EBV preventive strategies mainly involve avoidance of high-risk mismatches by matching a CMV or EBV seronegative recipient with a CMV or EBV seronegative donor, respectively. In living donor KT (LDKT), this would require the participation of donor-recipient pairs in kidney paired donation (KPD). For pairs that are HLA and ABO compatible, that is, ABO-HLA compatible pairs (CPs), this decision to participate in KPD will require balancing the potential benefits of avoiding high-risk CMV and EBV mismatches with the risks associated with delays in KT as well as logistical hurdles associated with KPD.^11,12^ Although the participation of CPs in KPD has generally been done for altruistic purposes, it has been suggested that KPD should provide additional benefits to the CP recipient (ie, improved matching for age, CMV, EBV, HLA).^11^ Published experience with the use of KPD for CMV and EBV matching is sparse.^13^ Additionally, little is known regarding interest in KPD among donors and recipients to avoid high-risk CMV and EBV mismatches. Prior studies evaluating KPD interest among CPs did not explore interest in avoiding CMV and EBV infections.^14^ This information is necessary before KPD can be explored as an option to avoid CMV/EBV high-risk mismatches. To address this gap, we present results from a survey of donor and kidney recipient candidates regarding their interest in KPD to mitigate risks from CMV and EBV infections.

## MATERIALS AND METHODS

### Cohort and Setting

This was a single-center cross-sectional survey study done at the University of Pittsburgh Medical Center (UPMC) between October 2022 and May 2023. Consecutive donors and potential kidney recipient candidates presenting for their initial evaluation clinic visit were recruited to participate in the study. Donors and recipient candidates were recruited separately for the study and were not required to be part of the same donor-recipient pair.

### Survey Development

A 5-point Likert choice-based survey was developed that asked participants to report their willingness to participate in KPD even if they were ABO and HLA compatible with their intended donor (or recipient). We modeled the survey questions on a prior study of altruistic donors and recipients from Ratner et al^14^ but added additional questions specifically pertaining to the willingness to participate in KPD to avoid CMV and EBV high-risk mismatched LDKT (see Full Survey in **Supplemental Digital Content, SDC,** http://links.lww.com/TXD/A719). The survey consisted of situations that offered an added advantage to the recipient, such as lower rejection risk, younger donor, lower CMV, and lower PTLD risks. The survey also evaluated willingness toward KPD in situations that did not offer an obvious added advantage to the intended recipient. Additionally, it assessed interest in situations pertaining to delays to KT due to KPD as well as logistics related to pairs being at different transplant centers. Response choices on the Likert scale were “strongly disagree,” “somewhat disagree,” “neither agree nor disagree,” “somewhat agree,” and “strongly agree.” To provide survey participants information on CMV and EBV-associated risks, we created an informational video that provided educational material on the following: (1) KPD process and uses, (2) ABO-HLA CPs, (3) CMV and EBV infections, (4) meaning of high-risk viral mismatches, and (5) risk of CMV infection and PTLD as well as complications including risk for graft failure and/or mortality that was derived from the published literature. Participants who agreed to participate in the study were required to watch this informational video before answering the survey questions. The initial survey and the information video were further modified on the basis of feedback from transplant clinicians (2 transplant nephrologists and 1 transplant surgeon within UPMC) and 4 nonclinician members (research faculty) within UPMC.

### Participant Recruitment and Survey Administration

All live donor candidates and KTR candidates presenting to UPMC were approached and recruited by a research coordinator who then administered the survey through the Qualtrics survey tool on portable electronic tablets. All participants were required to complete the survey during their initial evaluation (donor or recipient) clinic visits, after which the survey data were uploaded to the central Qualtrics data warehouse at the University of Pittsburgh. The research coordinator was available to assist with any technical or software issues during the survey completion. Our goal was to recruit at least 50 donors and 100 recipients in a 1:2 ratio, given the relatively higher number of recipient evaluations compared with donor evaluations.

### Data Analysis and Statistical Methods

Baseline characteristics of donors and recipients were summarized using descriptive statistics. Survey responses were analyzed to examine the following questions: (1) overall interest in KPD, (2) interest in KPD for CMV and EBV matching, (3) differences in donor and recipient candidate responses, (4) effect of transplant delays on KPD interest, (5) association between overall and altruistic KPD interest with interest in KPD for virological matching, and (6) association of key baseline characteristics with interest in KPD for altruistic paired exchange, for virological matching and with varying time delays to LDKT. We analyzed the Likert responses as a continuous outcome with differences evaluated using nonparametric tests (the Mann-Whitney *U* test for unpaired data and the Wilcoxon signed-rank test for paired analysis). Responses were also categorized as binary by combining “agree” and “strongly agree” as “favorable” responses. Analysis was performed with Stata version 17 (StataCorp, College Station, TX) and SAS version 9.4. (SAS Institute Inc, Cary, NC). A 2‐sided *P* value of <0.05 was considered statistically significant. The survey was approved by the University of Pittsburgh’s Institutional Review Board (STUDY22060108). All research activities were conducted according to the Declaration of Helsinki and the Declaration of Istanbul.

## RESULTS

### Survey Participants

Between October 2022 and March 2023, 51 donor and 102 recipient candidates who were approached for study participation agreed to participate in the study. None of the candidates declined participation. All survey responses were complete and had no missing data. Compared with the recipients, donors were younger (40 versus 59 y, *P* < 0.001), had a higher proportion of women (63% versus 33%, *P* < 0.001), had a higher proportion with college degrees (51% versus 28%, *P* = 0.004), had full-time employment (88% versus 27%, *P* < 0.001), and had higher annual household income (Table 1).

**TABLE 1.** Baseline characteristics among donor and recipient candidates

Characteristics	All (N = 153)	Donor (N = 51)	Recipient (N = 102)	*P*
Age, y, median (range)	53 (20–78)	40 (23–67)	59 (20–78)	<0.001
Sex, n (%)				0.001
Female	66 (43.1)	32 (62.7)	34 (33.3)	
Male	87 (56.9)	19 (37.3)	68 (66.7)	
Race, n (%)				<0.001
White	110 (71.2)	47 (92.2)	63 (61.8)	
Black	35 (23.1)	2 (3.9)	33 (32.4)	
Other	8 (5.2)	2 (3.9)	6 (5.9)	
Marital status, n (%)				0.149
Divorced	21 (13.7)	5 (9.8)	16 (15.7)	
Married	74 (48.4)	31 (60.8)	43 (42.2)	
Never married	46 (30.1)	13 (25.5)	33 (32.4)	
Separated or widowed	12 (7.8)	2 (3.9)	10 (9.8)	
Education, n (%)				0.004
High school graduate	47 (30.7)	8 (15.7)	39 (38.2)	
Some college but no degree/associate degree in college (2 y)	52 (34.0)	17 (33.3)	35 (34.3)	
Bachelor’s degree and above	54 (35.3)	26 (51.0)	28 (27.5)	
Household income, n (%)				<0.001
<$39 999	56 (39.1)	6 (11.7)	50 (54.3)	
$40 000–$99 999	57 (39.9)	23 (45.1)	34 (36.9)	
≥$100 000	30 (21.0)	22 (43.1)	8 (8.7)	
Job status, n (%)				<0.001
Not working	49 (34.0)	4 (7.8)	45 (48.4)	
Full time	70 (48.6)	45 (88.2)	25 (26.9)	
Part time	25 (17.4)	2 (3.9)	23 (24.7)	
Dialysis, n (%)				
No	–	–	41 (40.2)	
Yes	–	–	61 (59.8)	

### Interest in KPD

**FIGURE 1.**
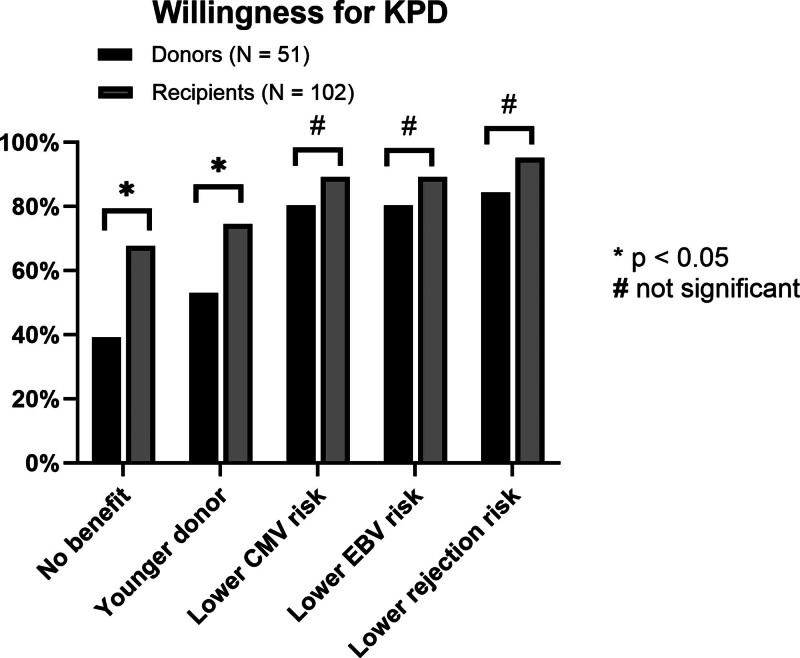
Interest in KPD participation for varying benefits to the recipient and under assumptions of donor-recipient ABO and HLA compatibility. In situations where donor-recipients were compatible, interest in KPD was lowest if there was no additional benefit to be gained from participating in KPD. Contrarily, interest in KPD participation was high among both donors and recipient candidates in situations that afforded added benefits to the recipient, such as reduced risk of CMV infection, EBV infection, and transplant rejection. CMV, cytomegalovirus; EBV, Epstein-Barr virus; KPD, kidney paired donation.

Overall interest in KPD was high and not different between donor and recipient candidates (median Likert score 4 versus 4, *P* = 0.09; Table 2). However, while recipient candidates had high interest in KPD irrespective of whether they were compatible with their donor or not, donor candidates expressed lower less interested in KPD if they were already compatible with their intended recipients (median Likert score 2 versus 4, *P* < 0.001; Table 2). Only 45.1% (N = 23) of donor candidates expressed interest in KPD if compatible compared with 80.4% (N = 82) of recipient candidates. Compared with recipient candidates, donor candidates were also less interested in KPD participation for getting a “younger donor” (median Likert score 4 versus 5, *P* = 0.008), and in situations where the recipient got “no additional benefit” (median score 3 versus 4, *P* = 0.006; Table 2; Figure 1).

**TABLE 2.** Interest in KPD among donor and recipient candidates under various circumstances

Interest in KPD	Full cohort, median (IQR)	Donor, median (IQR)	Recipient, median (IQR)	*P*
Overall interest in KPD	4 (3–4)	4 (3–4)	4 (3–5)	0.090
Interest in KPD if not a match with each other	4 (3–5)	4 (3–5)	4 (3–5)	0.399
Interest in KPD despite being a match with each other	3 (2–4)	2 (2–4)	4 (3–4)	<0.001
Scenarios with varying benefits				
If recipient gets additional benefit	4 (4–5)	4 (3–5)	4 (4–5)	0.943
Even if recipient gets no additional benefit	5 (4–5)	3 (2–5)	4 (3–5)	0.006
If recipient gets kidney from a younger donor	4 (3–5)	4 (3–5)	5 (4–5)	0.008
If it lowers recipient’s risk of kidney rejection	5 (4–5)	5 (4–5)	5 (4–5)	0.086
If it lowers recipient’s risk for CMV infection	5 (4–5)	5 (4–5)	5 (4–5)	0.120
If it lowers recipient’s risk for EBV-related lymphoma	5 (4–5)	5 (4–5)	5 (4–5)	0.166
Logistics of KPD				
If transplant had to be delayed by <1 mo	5 (4–5)	4 (4–5)	5 (4–5)	0.454
If transplant had to be delayed by 1–3 mo	4 (3–5)	4 (3–5)	4 (3–5)	0.078
If transplant had to be delayed by 3–6 mo	4 (3–5)	4 (2–5)	4 (3–5)	0.188
If the other pair is at the same transplant center	5 (3–5)	4 (3–5)	5 (4–5)	0.045
Even if the other pair is at a different transplant center	4 (3–5)	3 (3–5)	4 (3–5)	0.032

CMV, cytomegalovirus; EBV, Epstein-Barr virus; IQR, interquartile range; KPD, kidney paired donation.

### Interest in KPD for CMV and EBV Matching

Donor and recipient candidates expressed a similar degree of high willingness to participate in KPD for CMV and EBV matching (Table 2). Overall, 80.4% of donor candidates (N = 41) and 89.2% of recipient candidates (N = 91) expressed willingness (“agree” or “strongly agree”) for KPD to avoid high-risk CMV and EBV mismatches even if they were ABO-HLA compatible with their intended donor (or recipient; Figure 1). This interest in KPD for CMV and EBV matching was higher than in situations with no benefit to the recipients or situations with potential for younger donors (Figure 1). Among donor candidates, 52.9% (N = 27) expressed interest in KPD for “younger donors,” compared with 80.4% (N = 41) expressing interest in reducing CMV and EBV infections (*P* = 0.02). Among recipient candidates, these proportions were 74.5% (N = 76) and 89.2% (N = 91), respectively (*P* = 0.019).

### Influence of KPD Logistics on KPD Interest

**FIGURE 2.**
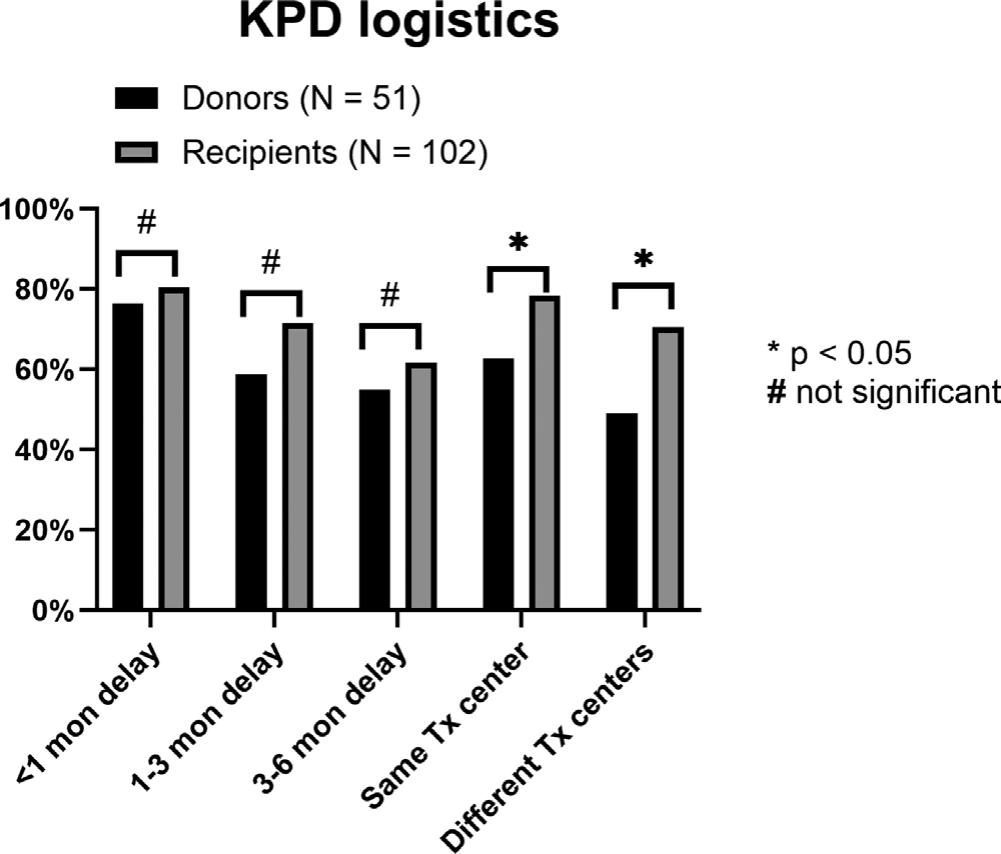
Influence of KPD logistics on interest in KPD participation. Among both donor and recipient candidates, interest in KPD was lower in situations with longer delays to transplantation. Participants, particularly donor candidates, were less enthusiastic about KPD if KPD involved pairs from other transplant centers. All responses pertain to situations that assume ABO and HLA compatibility among donors and recipients. KPD, kidney paired donation; Tx, transplant.

**FIGURE 3.**
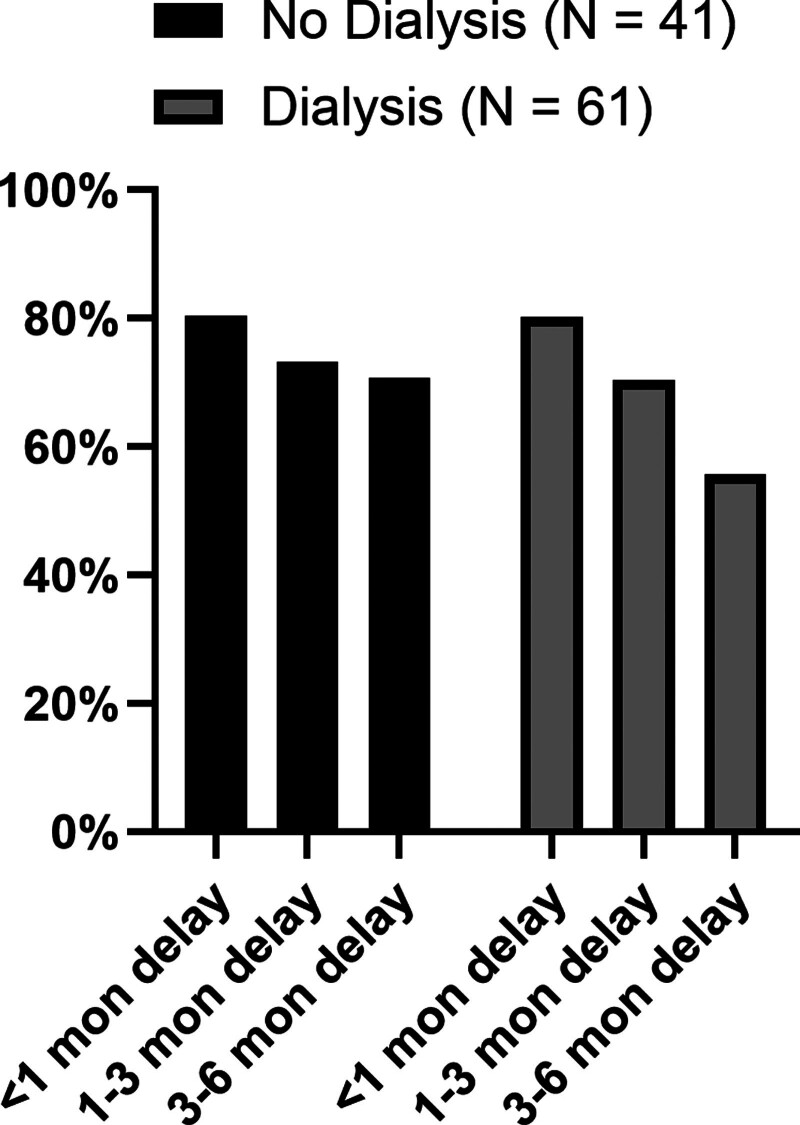
Recipient attitude to delays in transplant and interest in KPD based on dialysis status. Patients on dialysis viewed delays to transplantation less favorably than those not on dialysis with regard to KPD participation. All responses pertain to situations that assume ABO and HLA compatibility among donors and recipients. KPD, kidney paired donation

**FIGURE 4.**
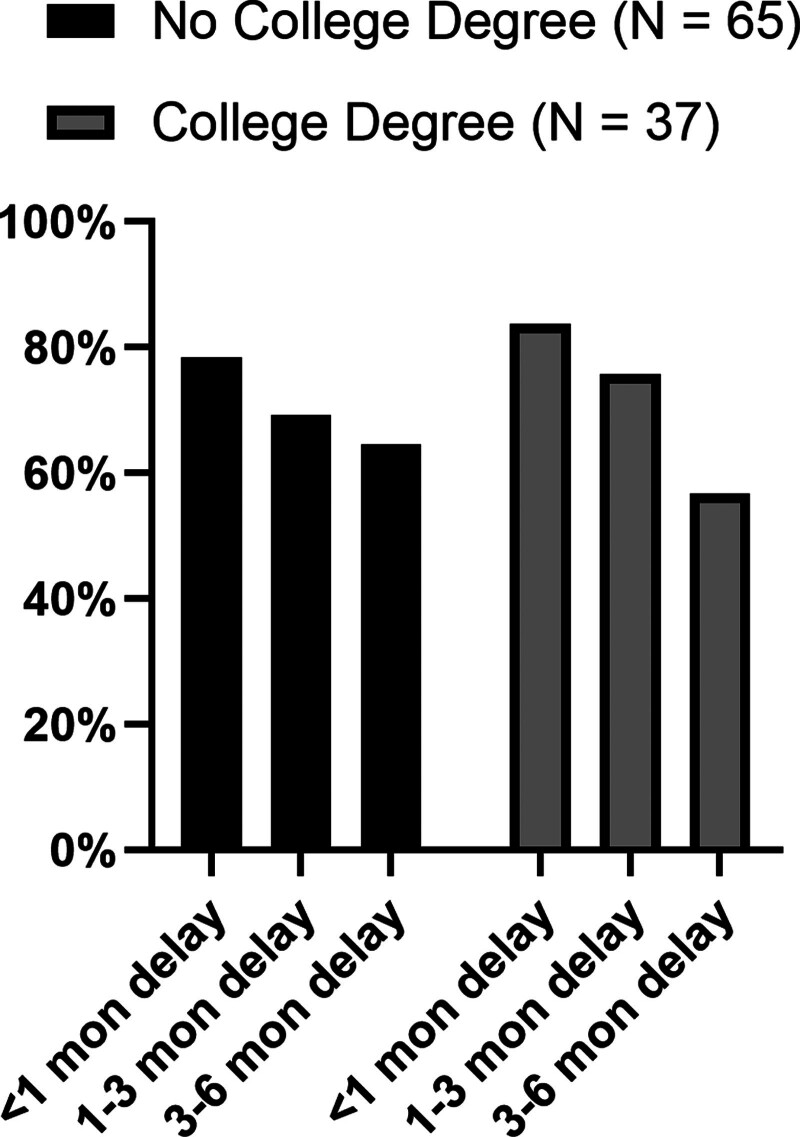
Recipient attitude to delays in transplant and interest in KPD based on education (college degree or not). Although recipient candidates with and without college degree both showed a lower preference for KPD as delays increased, the decrease in interest was steeper among those with college degrees. KPD, kidney paired donation

Respondents’ interest in KPD declined with increasing delays to KT. The proportion of candidates willing to participate in KPD at delays of <1, 1–3, and 3–6 mo were 79.1%, 67.3%, and 59.4%, respectively. This decreasing interest for KPD with longer delays to transplant was noted for both donor candidates (76.5%, 58.8%, and 54.9%; *P* < 0.001 for between-group comparisons) and recipient candidates (80.4%, 71.6%, and 61.8%; *P* < 0.001 for between-group comparisons (Figure 2). Additionally, both donors and recipients indicated higher willingness for KPD if the pairs were within the same transplant center than if the KPD pairs were at different transplant centers (full cohort, 73.2% versus 63.4%, *P* < 0.001; Table 2; Figure 2). Furthermore, among the 2 groups, donor candidates were less enthusiastic about KPD than recipient candidates if pairs were at different transplant centers (49% versus 70.6% for donor and recipient candidates, respectively; *P* = 0.009; Figure 2). When the influence of transplant delays on KPD interest was evaluated among recipient candidates with and without dialysis, no statistically significant difference was noted. However, there was a suggestion that if delays were >3 mo, a smaller proportion of candidates on dialysis expressed interest in KPD compared with candidates who were not on dialysis (55.7% versus 70.7%, *P* = 0.13; Figure 3). Similar trends were observed among recipients with and without college degrees, with interest in KPD declining more sharply among those with college degrees compared with those without (Figure 4).

### Baseline Characteristics and Interest in KPD

Baseline characteristics did not influence interest in KPD for CMV matching or EBV matching and reduce risk of rejections potentially. However, differences were noted in other aspects of KPD. Black candidates expressed higher interest in KPD than non-Black candidates if the KPD pairs were at different transplant centers (80% versus 58%, *P* = 0.019). Recipient candidates with college degrees expressed less interest in KPD if there was no added benefit (45.7% versus 68.7%, *P* = 0.004). Recipient candidates who were working part time or full time had a lower overall interest in KPD (84.2% versus 95.9%, *P* = 0.039), particularly if they were already compatible with their donors (61.1% versus 83.7%, *P* = 0.006). Also, recipient candidates in the higher income category (≥$100 000) had a lower interest in KPD compared with candidates with an annual income of <$40 000 and $40–<$100 000 if they were compatible with their donor (50% versus 88% versus 82.4%, *P* = 0.031), if there was no added benefit (25% versus 74% versus 70.6%, *P* = 0.02), and if the KPD pairs were at different centers (25% versus 78% versus 70.6%, *P* = 0.009). There was no evidence of an association between baseline characteristics and survey responses among donor candidates.

## DISCUSSION

Donor exchanges through KPD offer a potential option for reducing high-risk donor-recipient mismatches to CMV and EBV. There, however, remains a gap in our understanding regarding the interest in KPD among potential kidney donors and recipients to avoid high-risk CMV and EBV mismatches. It also remains unclear whether this interest varies among donor and recipient candidates and whether donor and recipient candidate characteristics influence interest in KPD. In this cross-sectional survey of prospective donor and recipient candidates, we show that the interest in KPD to avoid high-risk mismatches to CMV and EBV was high among both donor and recipient candidates. Interest in KPD, however, waned with longer delays to transplantation and when KPDs involved external transplant centers.

Several findings from this study merit further discussion. In general, the high interest in KPD to avoid high-risk CMV and EBV mismatches is noteworthy. The level of interest was similar to the participants’ interest in KPD to reduce the risk of rejection. Although there has been a recent increase in interest in matching donor and recipients based on HLA,^15-17^ there has been limited exploration in using KPD to avoid high-risk CMV and EBV mismatches. Given the negative consequences associated with donor-derived CMV and EBV infections, it is imperative to also assess LDKT pairs from a virological mismatch standpoint.

Incorporating CMV and EBV as potential matching variables when considering KPD for CPs will require several issues. Most importantly, KPDs are associated with delays to transplantation, and donors and recipients are wary of these delays, as demonstrated by the lower interest in KPD in our study when delays extended beyond 3 mo. Although the benefits of matching may still outweigh the risks associated with waiting, the maximum amount of time that recipients would wait has not been clearly established. A simulation model for CMV matching in deceased donor KT compared mortality among waitlisted patients to survival benefits of avoiding high-risk CMV.^18^ This simulation model found that waiting up to 30 mo was superior to transplanting with a high-risk CMV mismatch,^18^ but additional work is needed because this model incorporated data from only deceased donor KT and not LDKT. Similar work on EBV is lacking. This is of particular importance for EBV given the low EBV seronegative status (approximately 8%–10%) among potential donors,^19,20^ compared with 30%–40% seronegative status among donors for CMV.^21,22^ It is possible that the amount of time patients could safely wait may vary based on the patient subgroups and the risk of poor outcomes while awaiting a CMV/EBV-matched donor.

Despite the potential for longer allograft and patient survival by avoiding high-risk CMV/EBV mismatches, both donors and recipients themselves may not prioritize waiting over timely transplantation. This may relate to low quality-of-life issues on dialysis (for recipients) and to issues related to interference with education, work, and other activities (for donors). We observe that as delays to transplantation increased, there was a suggestion of decreased KPD interest among patients who were on dialysis compared with those who were not.

In addition, respondents were more interested in KPD if the pairs were from the same transplant center rather than at different centers. The reasons for this are not entirely clear but may be related to concerns regarding kidney transportation, disruptions in KPD chains, perceived convenience and trust in their own transplant center, and concerns about potential delays, unfamiliarity, and quality of care at different centers. Considering that the likelihood of finding a suitable match is higher with multicenter/national KPD programs rather than single-center KPDs, potential pairs will need to be provided with appropriate education regarding the KPD process, thus allowing more willing pairs to participate in national KPDs to improve their matching opportunities.

Our study suggests that the interest in KPD, even when donors and recipients are ABO-HLA compatible, is quite high. Because CPs do not necessarily have to participate in KPD, it has been proposed that providing additional benefits to the recipient of a CP may increase KPD participation.^12,23,24^ It is important to note that while the donor candidates reported lower interest in KPD when there was no added benefit, they expressed more interest in KPD in situations that afforded additional benefit, such as lower risk of CMV, EBV, or rejection for the recipient. Donor candidates in our study had higher levels of education, were more likely to be employed, and had higher median income; thus, given the logistical challenges associated with KPD, the extra waiting and its interference with their job and social activities may explain their reluctance to participate in KPD in situations that provide no additional benefit. These same reasons may explain the lower interest in KPD among donors in situations involving CPs and KPDs that involved multiple transplant centers.

Despite the potential for improving outcomes in LDKT, KPD for CPs has been used sparingly. Reasons for this are unknown but may relate to ethical concerns,^12,14,25^ logistical challenges of KPD, and lack of evidence regarding the feasibility of using KPD for virological matching. By participating in KPD and expanding the pool size, high-risk CMV/EBV CPs could increase LDKT access for other mismatched pairs awaiting LDKT through KPD.^26^ Simulation modeling^24^ suggests that even among single-center KPDs, the entry of CPs can double the match rates for other incompatible pairs. Thus, there exists the potential to increase LDKT numbers and improve upon the recently noted KPD-driven increase in LDKT.^27^ However, such expansion of KPD through CP participation will require a structured process to ensure that prospective donors, recipients, and CPs are educated and informed of the risks and benefits of KPD. Given that there are multiple domains of donor-recipient mismatches (age, body size, HLA, CMV, EBV), there remains uncertainty on what should be prioritized. Hence, additional work in this area is needed, including easily available information on details of potential waiting times based on match requirements and donor and recipient characteristics. From a practical perspective, for CPs, care should be taken to not worsen donor-recipient mismatches if possible and a time-limited trial of KPD with frequent discussions and confirmation regarding the CPs interest to continue in KPD should be considered. Additionally, the potential for prolonged cold ischemia time should also be considered, particularly with KPDs involving multiple centers. This is important because studies suggest worse LDKT when cold ischemia times exceed 4–8 h.^28^ Finally, it is unclear whether the expenses related to the use of national KPD programs will be a barrier for centers to enter their CPs into KPDs.

The study findings must be considered in light of the following limitations. First, our study cohort was drawn from a single center, and there was an underrepresentation of racial and ethnic minorities, particularly among donor candidates. As a result, the findings may not be generalizable to populations that differ from our study’s racial and ethnic composition. We did not evaluate participants’ understanding of the information provided on CMV and EBV-related risks at the beginning of the survey. We did not examine the reasons why patents expressed lower interest in some situations. The study also did not examine how donors and recipients would prioritize attributes related to LDKT and KPD, such as time to LDKT, CMV infection, PTLD, graft and patient survival after LDKT, etc. We also did not assess the magnitude of risk (for CMV, PTLD, etc) and the tradeoffs that participants were willing to make (eg, whether they would be willing to wait for a longer time in some scenarios) when considering KPD over direct donation. Despite these limitations, our study provides useful data regarding interest in KPD for CMV and EBV matching, which has not been previously examined. Another strength of the study includes the examination of variation in KPD interest among donors and recipients in circumstances with varied benefits to the recipient.

There are several key findings from the study that clinicians should consider when evaluating living donor and recipient pairs. Although KPD is typically offered in cases of ABO and/or HLA incompatibility, the study suggests that clinicians should also consider offering KPD to ABO-HLA CPs. It is essential to discuss the risks, particularly the anticipated delays, as well as the potential to improve recipient outcomes by avoiding high-risk (deleterious) donor-recipient mismatches. Although donors and recipients were less enthusiastic about using external KPDs, providing education about the limitations and benefits of both within-center and multicenter/national KPDs is crucial to ensure that all potential KPD options are explored to minimize delays and improve the chances of finding suitable match. The study also suggests that patient characteristics, such as employment status and level of education, influence decision-making, highlighting the need to explore patient-specific (donor) barriers and concerns. Finally, care should be taken to ensure that the process is conducted in a noncoercive manner, with full disclosure of risks and benefits.

In conclusion, using a cross-sectional survey, we show that prospective kidney donor and recipient candidates have a high interest in KPD participation to avoid high-risk CMV and EBV mismatches. We also show that donors have lower interest in participating as CPs in KPD when there are no additional benefits offered. Finally, both donor and recipient candidates had concerns about delays to transplant and participation in KPDs that involve multiple transplant centers. Collectively, these findings provide impetus for further studies to explore the expansion of KPD among ABO-HLA CPs.

## Supplementary Material


