# Supplementary material for: Mitigating High-risk EBV and CMV Through Kidney Paired Donation: A Survey of Potential Donor and Recipient Candidates
Source: Transplant Direct. 2024 Nov 15;10(12):e1737. doi: 10.1097/TXD.0000000000001737 (PMC11576024; doi:10.1097/TXD.0000000000001737)
Supplement: Supplementary file 1 [file txd-10-e1737-s001.pdf]

SUPPLEMENTAL DIGITAL CONTENT (SDC)

Table of contents

Table of contents	Page (s)
**SDC, Materials and Methods:** KPD interest Recipient survey- full format	2-15

Kidney Paired Donation - Recipient Survey

Start of Block: Informed Consent

Q1 Hello ! Thank you for your interest in this research study on Kidney Paired Donation (KPD)

This survey consists of a brief video about kidney paired donation followed by questions regarding your interest in kidney paired donation. Altogether, this should take about 10-15 minutes.

Your responses will be collected and stored anonymously. The risks of participating in this study are minimal. Your participation is voluntary and you have the right to withdraw from the study at any point.

This study has been approved by the University of Pittsburgh's IRB. Please contact the study investigator Dr. Puttarajappa (email: puttarajappacm@upmc.edu) if you have any questions.

By clicking the button below, you acknowledge that:
Your participation in the study is voluntary
You are 18 years of age.

○ I consent, begin the study

○ I do not consent, I do not wish to participate

End of Block: Informed Consent

Start of Block: Block 1

Q2 **Please click the video link below to watch the video. Please return to the survey after watching the video.**

Video link

Q3 **The next few questions will ask about your interest and willingness regarding donor exchanges through KPD, specifically in situations where <u>you may already be a match</u> with your potential kidney donor and thus <u>*do not necessarily need to*</u> participate in KPD.**

Please answer these questions even if you currently do not have any potential live donor candidates.

Q4 **Assuming that you are <u>already a match with your kidney donor and thus do not necessarily need to participate in KPD</u>, please indicate your interest regarding donor exchanges through KPD in the following situations**

	Strongly disagree	Somewhat disagree	Neither agree nor disagree	Somewhat agree	Strongly agree
I will participate if I get some additional benefit	○	○	○	○	○
I will participate if I get a kidney from a younger donor	○	○	○	○	○
I will participate even if I get no additional benefit	○	○	○	○	○
I will participate if it lowers my risk of kidney rejection	○	○	○	○	○
I will participate if it lowers my risk for cytomegalovirus (CMV) infection	○	○	○	○	○
I will participate if it lowers my risk for Epstein-Barr virus (EBV) related lymphoma (cancer)	○	○	○	○	○

Q5 **Assuming that you are already a match with your kidney donor and thus do not necessarily need to participate in KPD, please indicate your interest regarding donor exchanges through KPD in the following situations**

	Strongly disagree	Somewhat disagree	Neither agree nor disagree	Somewhat agree	Strongly agree
I will participate if it helps another recipient	○	○	○	○	○
I will participate if the other recipient is known to me	○	○	○	○	○
I will participate if the other recipient is my relative	○	○	○	○	○
I will participate if the other recipient is a child	○	○	○	○	○
I will participate even if the other recipient is not known to me	○	○	○	○	○
I will participate if my intended donor is also interested to participate	○	○	○	○	○

Q6 **Assuming that you are <u>already a match with your kidney donor and thus do not necessarily need to participate in KPD</u>, please indicate your interest regarding donor exchanges through KPD in the following situations**

	Strongly disagree	Somewhat disagree	Neither agree nor disagree	Somewhat agree	Strongly agree
I will participate in KPD if the other donor-recipient pair is at the same transplant center as mine	○	○	○	○	○
I will participate in KPD even if the other donor-recipient pair is at a different transplant center than mine	○	○	○	○	○
Participation in KPD will be an added stress to me	○	○	○	○	○
Decision to participate in KPD should be a mutual decision between the donor and the recipient	○	○	○	○	○

Q7 **Assuming that you are already a match with your kidney donor and could proceed to transplant without any delay, please indicate whether you would participate in donor exchanges through KPD in the following situations**

	Strongly disagree	Somewhat disagree	Neither agree nor disagree	Somewhat agree	Strongly agree
I will participate if transplant had to be delayed by less than 1 month	○	○	○	○	○
I will participate even if transplant had to be delayed by an additional 1-3 months	○	○	○	○	○
I will participate even if transplant had to be delayed by an additional 3-6 months	○	○	○	○	○

End of Block: Block 1

Start of Block: Block 5

Q8 **The next three questions will ask about your general interest regarding KPD participation**

End of Block: Block 5

Start of Block: Matrix questions

Q9 **Assuming that you are already a match with your kidney donor and thus do not necessarily need to participate in KPD, please indicate whether you would still be interested in donor exchanges through KPD**

- ○ Not interested at all
- ○ Slightly interested
- ○ Moderately interested
- ○ Very interested
- ○ Extremely interested

Q10 **In situations where your kidney donor is not a match for you and thus cannot directly donate a kidney to you, please indicate whether you would be interested in donor exchanges through KPD.**

- ○ Not interested at all
- ○ Slightly interested
- ○ Moderately interested
- ○ Very interested
- ○ Extremely interested

Q11 **Please indicate your overall interest for KPD participation**

- ○ Not interested at all
- ○ Slightly interested
- ○ Moderately interested
- ○ Very interested
- ○ Extremely interested

Q12 **The next set of questions will ask for information about you**

End of Block: Matrix questions

Start of Block: Block 4

Q13 **Are you currently on dialysis?**

- ○ Yes
- ○ No

Q14 **For how many years have you been on dialysis?**

- Less than a year
- 1 year
- 2 years
- 3 years
- 4 years
- 5 years or more

Q15 **What type of dialysis are you currently undergoing?**

- Hemodialysis at a dialysis center
- Peritoneal dialysis
- Home hemodialysis

Page Break

Q16 **Has anyone expressed interest in being a live kidney donor to you?**

- ○ Yes
- ○ No
- ○ I have not asked anyone yet
- ○ I am not comfortable asking a living person to donate a kidney to me

End of Block: Block 4

Start of Block: Demographics

Page Break

*

Q17 **What is your YEAR of birth? (Please enter only your 4 digit year of birth)**

Q18 **What is the highest level of school you have completed or the highest degree you have received?**

- ○ Less than high school degree
- ○ High school graduate (high school diploma or equivalent including GED)
- ○ Some college but no degree
- ○ Associate degree in college (2-year)
- ○ Bachelor's degree in college (4-year)
- ○ Master's degree
- ○ Doctoral degree
- ○ Professional degree (JD, MD)

Q19 **Are you Spanish, Hispanic, or Latino or none of these?**

- ○ Yes
- ○ None of these

Q20 **Choose one or more races that you consider yourself to be:**

- ☐ White
- ☐ Black or African American
- ☐ American Indian or Alaska Native
- ☐ Asian
- ☐ Native Hawaiian or Pacific Islander
- ☐ Other ____

Q21 **Which of the following most likely describes you?**

- Male
- Female
- Transgender
- Non-binary
- Prefer not to say

Q22 **Are you now married, widowed, divorced, separated or never married?**

- Married
- Widowed
- Divorced
- Separated
- Never Married

Q23 **Information about income is important to understand. Would you please give your best guess? Please indicate the answer that includes your entire household income in (previous year) before taxes.**

- Less than $20,000
- $20,000 to $39,999
- $40,000 to $69,999
- $70,000 to $99,999
- $100,000 to $149,999
- $150,000 or more
- Prefer not to say

Q24 **Which statement best describes your current employment status?**

- ○ Working full time (paid employee)
- ○ Working full time (self-employed)
- ○ Working part time
- ○ Not working (temporary layoff from a job)
- ○ Not working (looking for work)
- ○ Not working (retired)
- ○ Not working (disabled)
- ○ Not working (other) ____
- ○ Prefer not to say

*

Q25 **What is your ZIP code?**

____

End of Block: Demographics
